# Mutations of the *BRCA1* and *BRCA2* genes in patients with bilateral breast cancer

**DOI:** 10.1054/bjoc.2001.2016

**Published:** 2001-09

**Authors:** D Steinmann, M Bremer, D Rades, B Skawran, C Siebrands, J H Karstens, T Dörk

**Affiliations:** Department of Radiation Oncology, Institute of Human Genetics, Department of Biochemistry and Tumour Biology, Clinic of Obstetrics and Gynecology, Medical School Hannover, Hannover, Germany

**Keywords:** contralateral breast cancer, *BRCA1*, *BRCA2*

## Abstract

Mutations of the *BRCA1* or *BRCA2* genes have been shown to strongly predispose towards the development of contralateral breast cancer in patients from large multi-case families. In order to test the hypothesis that *BRCA1* and *BRCA2* mutations are more frequent in patients with bilateral breast cancer, we have investigated a hospital-based series of 75 consecutive patients with bilateral breast cancer and a comparison group of 75 patients with unilateral breast cancer, pairwise matched by age and family history, for mutations in the *BRCA1* and *BRCA2* genes. Five frameshift deletions (517delGT in BRCA1; 4772delA, 5946delCT, 6174delT and 8138del5 in BRCA2) were identified in patients with bilateral disease. No further mutations, apart from polymorphisms and 3 rare unclassified variants, were found after scanning the whole *BRCA1* and *BRCA2* coding sequence. Three pathogenic *BRCA1* mutations (Cys61Gly, 3814del5, 5382insC) were identified in the group of patients with unilateral breast cancer. The frequencies of common *BRCA1* and *BRCA2* missense variants were not different between the 2 groups. In summary, we did not find a significantly increased prevalence of *BRCA1* and *BRCA2* mutations in a hospital-based cohort of German patients with bilateral breast cancer. We conclude that bilaterality of breast cancer on its own is not strongly associated with *BRCA1* and *BRCA2* mutations when adjusted for age and family history. The high frequency of bilateral disease in multi-case breast cancer families may be due to a familial aggregation of additional susceptibility factors modifying the penetrance of *BRCA1* and *BRCA2* mutations. © 2001 Cancer Research Campaignhttp://www.bjcancer.com


*British Journal of Cancer* (2001) **85**(6), 850–858
© 2001 Cancer Research Campaign
doi: 10.1054/ bjoc.2001.2016, available online at http://www.idealibrary.com on IDEAL

### Mutations of the *BRCA1* and *BRCA2* genes in patients with bilateral breast cancer

**D Steinmann[1,2]*, M Bremer[1], D Rades[1], B Skawran[2], C Siebrands[2], JH Karstens[1] and T Dörk[2,3]**

[1]Department of Radiation Oncology, [2]Institute of Human Genetics, and [3]Department of Biochemistry and Tumour Biology, Clinic of Obstetrics and Gynecology, Medical School Hannover, Hannover, Germany

**Summary** Mutations of the *BRCA1* or *BRCA2* genes have been shown to strongly predispose towards the development of contralateral breast cancer in patients from large multi-case families. In order to test the hypothesis that *BRCA1* and *BRCA2* mutations are more frequent in patients with bilateral breast cancer, we have investigated a hospital-based series of 75 consecutive patients with bilateral breast cancer and a comparison group of 75 patients with unilateral breast cancer, pairwise matched by age and family history, for mutations in the *BRCA1* and *BRCA2* genes. Five frameshift deletions (517delGT in BRCA1; 4772delA, 5946delCT, 6174delT and 8138del5 in BRCA2) were identified in patients with bilateral disease. No further mutations, apart from polymorphisms and 3 rare unclassified variants, were found after scanning the whole *BRCA1* and *BRCA2* coding sequence. Three pathogenic *BRCA1* mutations (Cys61Gly, 3814del5, 5382insC) were identified in the group of patients with unilateral breast cancer. The frequencies of common *BRCA1* and *BRCA2* missense variants were not different between the 2 groups. In summary, we did not find a significantly increased prevalence of *BRCA1* and *BRCA2* mutations in a hospital-based cohort of German patients with bilateral breast cancer. We conclude that bilaterality of breast cancer on its own is not strongly associated with *BRCA1* and *BRCA2* mutations when adjusted for age and family history. The high frequency of bilateral disease in multi-case breast cancer families may be due to a familial aggregation of additional susceptibility factors modifying the penetrance of *BRCA1* and *BRCA2* mutations. © 2001 Cancer Research Campaign http://www.bjcancer.com

**Keywords**: contralateral breast cancer; *BRCA1*; *BRCA2*

About 1 in 9 women in European and North American populations may develop breast cancer throughout their life-time, with rates having increased during the last decades (Philips et al, 1999). Each year there are approximately 46 000 new cases in Germany (Beckmann et al, 1997). Several risk factors have been defined including age, family history, smoking, hormonal factors and radiation exposure (Goss and Sierra, 1998; Olsen et al, 1999; Philips et al, 1999; Martin and Weber, 2000). Familiar predisposition accounts for some 10% of breast cancer, and about half of these patients are thought to have a mutation in one of two large genes that function in DNA damage response, *BRCA1* and *BRCA2* (Kote-Jarai and Eeles, 1999; Martin and Weber, 2000).

Bilaterality of cancer, including breast carcinoma, is thought to be an indicator of a genetic predisposition (Anderson, 1971; Knudson, 1971), and studies of blood relatives and of twins affected by breast cancer have corroborated this view (Bernstein et al, 1992; Peto and Mack, 2000). Contralateral breast cancer accounts for about 2–10% of patients (Hislop et al, 1984; Adami et al, 1985; Healey et al, 1993; Mose et al, 1995) and is observed at an annual incidence of approximately 0.7% per year (Peto and Mack, 2000). The importance of *BRCA1* and *BRCA2* for the bilateral occurrence of breast cancer has been demonstrated in large multi-case families where heterozygosity for a *BRCA1* or *BRCA2* gene mutation strongly predisposes the affected patients towards the development of a contralateral breast cancer (Ford et al, 1994, 1998; Easton et al, 1995; Robson et al, 1998; Verhoog et al, 1998). Young age at onset appears to be a significant risk factor for contralateral breast cancer in the general population (Hislop et al, 1984; Adami et al, 1985) and also within *BRCA1* families, where the risk declines for patients with a postmenopausal primary tumour (Verhoog et al, 2000). Initial population-based studies of selected *BRCA1* and *BRCA2* mutations have provided first evidence for an apparently low proportion of mutation carriers among patients with bilateral breast cancer when their age at first onset was higher than 40 years (Eccles et al, 1998; Gershoni-Baruch et al, 1999). As an attempt to determine more closely the relative contribution of *BRCA1* and *BRCA2* mutations to bilateral breast cancer in relation to age and family history, we have performed a hospital-based study of the whole coding region of the *BRCA1* and *BRCA2* genes in 75 German patients with bilateral breast cancer, and compared the results with the prevalence of *BRCA1* and *BRCA2* mutations in a pairwise-matched cohort of patients with unilateral breast cancer.

### PATIENTS AND METHODS

### Patients

During the years 1996–2000, more than 1000 unselected patients with breast cancer received radiotherapy in the Department of Radiation Oncology of the Medical School Hannover. The patients were counselled about the possibility of a genetic predisposition of breast cancer, and anamneses were taken using questionnaires with regard to the family history and previous radiation exposure

*Received 15 February 2001*
*Revised 30 May 2001*
*Accepted 11 June 2001*

*Correspondence to:* T Dörk

* Present address: Department of Haematology and Oncology, Medical School Hannover, Hannover, Germany.

of the patients. After written informed consent had been obtained, blood samples were collected from 1000 participating patients. 75 patients had developed bilateral breast cancer and took part in this study. This proportion of 7.5% of cases with bilateral disease was the same as has been previously reported in another series of over 500 German breast cancer patients (Mose et al, 1995), indicating that our cohort is representative for a hospital-based cancer population in Germany. As a comparison group for mutation frequency determination, we selected 75 patients with unilateral breast cancer from the total cohort who were pairwise matched with respect to age and family history at the time of treatment.

Clinical data of the patients with bilateral breast cancer were collected and compared with data of the whole series of 1000 breast cancer patients. In 29 patients, bilateral breast cancer occurred simultaneously. In the other 46 patients, contralateral breast cancer developed with a median time interval of 7.2 years (range 1 to 40 years). A time interval of at least 3 years was observed in 37 patients. The median age at diagnosis of the first breast cancer was 54 years, compared with 57 years in the total cohort. 22 patients (29.3%) were younger than 50 years and 9 patients (12%) younger than 40 years at first diagnosis. Self-reported family history revealed at least one first or second degree relative with breast cancer in 17 (22.7%) patients and ovarian cancer in 1 (1.3%) patient, which was not different from the figures obtained for the total group of unselected breast cancer patients ($n$ = 1000).

Breast conservative surgery of the first and second tumour was performed in 48 (64%) and 65 (86.7%) patients, respectively. Postoperative radiotherapy was applicated unilaterally in 18 patients (24%) and bilaterally in 57 patients (76%) with a median total dose of 50 Gy (single dose: 1.8 Gy). Of the latter group 18 patients received radiotherapy simultaneously to both sides without overlap of treatment fields. The median follow up for the first and second tumour was 6.3 and 2.5 years, respectively. 10 patients developed local (one patient bilaterally), 1 patient regional (axillary node) and 1 patient locoregional recurrence after a median time interval of 24 months. It is noteworthy that 3 of 6 (50%) identified carriers of a pathogenic *BRCA1* or *2* mutation showed local recurrence, whereas this was the case in only 9 of 69 noncarriers (13%). The actuarial locoregional relapse-free survival – as evaluated by Kaplan–Meier calculations – was significantly lower (log-rank test, $P$ = 0.0072) for carriers (mean: 55 months, 95% CI: 36–74 months) compared to non-carriers (mean: 408 months, 95% CI: 354–462 months). Local relapse after initial radiotherapy occurred in 5 patients (5 of 126 irradiated breasts). 3 of these 5 patients (60%) were carriers of a pathogenic *BRCA1* or *BRCA2* mutation. Distant metastases developed in 14 patients (18.7%) with 10 patients having died of their disease at time of analysis.

### Methods

EDTA blood samples from peripheral venous blood were collected from the patients of both groups, and genomic DNA was extracted from white blood cells according to a standard salting out procedure. PCR conditions were established to separately amplify all coding exons and exon-flanking intron sequences of both, *BRCA1* and *BRCA2* genes to investigate the samples obtained from patients with bilateral breast cancer (Appendix, Table 1). We designed a set of 26 primer pairs for the *BRCA1* gene and 29 primer pairs for the *BRCA2* gene based on the published genomic sequence (GenBank Acc. No. L78833 for the *BRCA1* gene, Z74739 for the *BRCA2* gene). Due to their large size, the coding sequences of exon 11 of the *BRCA1* gene (3.4 kb) and exon 11 of the BRCA2 gene (4.9 kb) were amplified in overlapping fragments. On the other hand, exons 5 and 6 and exons 23 and 24 of the *BRCA2* gene could be jointly amplified together with their intervening intron sequences.

Standard PCR amplifications were performed in 20 µl reaction volumes containing 100–200 ng of genomic DNA, 0.2 mM of each dNTP and 1 Unit Taq DNA polymerase in 1 × reaction buffer supplied by the manufacturer (USB/Amersham). Final primer concentrations were 0.5 µM. As the standard procedure, 37 PCR cycles were performed with 1 min denaturation at 94˚C, 1 min annealing, and 1 min elongation at 72˚C. PCR products over 1 kbp were amplified at an elongation time of 100–150 s, and in 2 cases (primer pairs BS13/14 and BS15/16) the reaction mix was supplemented with 0.1 M betaine and 2% formamide. Some exons could be amplified and analysed in a duplex PCR format (Appendix, Table 2).

Restriction fragments of 150 to 400 bp (Appendix, Table 3) were subjected to single strand conformation polymorphism (SSCP) analysis. In brief, the products were separated on a 40 cm 5% non-denaturating polyacrylamide gel at 40 watts for 5 h in a cold room, and were subsequently visualized by conventional silver staining. Aberrantly migrating samples were sequenced on both strands using an ABI-dRhodamine Terminator Cycle Sequence Kit, and sequencing data were collected and evaluated on an ABI 310 Genetic Analyzer (Perkin Elmer). Identified variants were then screened by restriction enzyme-based assays (Appendix, Table 4a) in both groups of patients. Some tests required a mismatch primer to create recognition sites for the respective restriction enzymes (Appendix, Table 4b). In addition, the common mutations 5382insC and Cys61Gly in the *BRCA1* gene and 6174delT in the *BRCA2* gene were analysed by restriction enzyme analysis in all patients, and a subset of patients in the comparison group who had a positive family history of breast cancer were scanned for mutations in the large exons of *BRCA1* (exon 11) and *BRCA2* (exons 10 and 11).

Statistical evaluation was performed using two-sided chi-square tests. The allele frequencies of truncating mutations were compared between both groups and results were considered to be significant for $P < 0.05$. In addition, the frequencies of missense substitutions that were found more than once, were compared between both cohorts and results and were considered to be significant if $P < 0.004$, following Bonferroni's correction for multiple testing with a number of 12 independent, i.e. unlinked, common substitutions.

### RESULTS

All coding exons and flanking intronic regions of the *BRCA1* and *BRCA2* genes were amplified by PCR from genomic DNA samples of 75 patients with bilateral breast cancer and were investigated by single-strand conformation polymorphism (SSCP) and sequencing analysis. Detected sequence alterations were subsequently confirmed by restriction enzyme analyses and screened in the comparison cohort of 75 pairwise-matched patients with unilateral breast cancer. In addition, a screening of the 3 most common pathogenic mutations as well as a mutation scanning of the largest exons of the *BRCA1* and *BRCA2* genes was also performed in the comparison cohort (see Patients and Methods

**Table 1** Mutations of the *BRCA1* and *BRCA2* genes in patients with bilateral and unilateral breast cancer

| Gene | Exon | Mutation | Predicted effect | Patient no. (status) | |
|---|---|---|---|---|---|
| | | | | Bilateral | Unilateral |
| *BRCA1* | 5 | T300G | Cys→Gly at 61 | | 1 het |
| | 7 | 517delTG | Truncation | 1 het | |
| | 11 | 3814del5 | Truncation | | 1 het |
| | 20 | 5382insC | Truncation | | 1 het |
| *BRCA2* | 11 | 4772delA | Truncation | 2 het(*) | |
| | 11 | 5946delCT | Truncation | 1 het | |
| | 11 | 6174delT | Truncation | 1 het | |
| | 17 | 8138del5 | Truncation | 1 het | |

Identified mutations are designated according to their nucleotide position within the BRCA1 or BRCA2 cDNA sequences. het: heterozygote. (*): two 4772delA heterozygous patients were mother and daughter, respectively.

section). A total of 8 different pathogenic *BRCA1* and *BRCA2* gene mutations were identified, with 5 heterozygous carriers among the patients with bilateral breast cancer and 3 heterozygous carriers among the patients with unilateral breast cancer (Table 1). All 5 mutations of the patients with bilateral breast cancer were small frameshift deletions of 1–5 basepairs leading to a premature translation termination codon. 4 truncating mutations (5946delCT, 6174delT, 8138del5, and the new 4772delA mutation) were found in the *BRCA2* gene, and one frameshift deletion (517delTG) was uncovered in the *BRCA1* gene (Figure 1, Table 1). The mutations in the group of patients with unilateral breast cancer were all in *BRCA1* and include the Cys61Gly substitution of the RING finger motif, the 5382insC insertion, and a 5-basepair deletion, 3814del5 (Figure 1, Table 1). There was no concordant occurrence of *BRCA1* or *BRCA2* mutations in paired samples of our matched cohorts. The 3814del5 mutation in the *BRCA1* gene and the 4772delA mutation in the *BRCA2* gene may be confined to the single families described here since they had not been reported previously to the Breast cancer Information Core (BIC) database (http://www.nhgri.nih.gov/Intramural_research/Lab_transfer/BIC/).

The clinical characteristics of the mutation carriers are summarized in Table 2. In the study cohort of patients with bilateral cancer, one carrier of the 6174delT mutation in the *BRCA2* gene had developed a synchronous bilateral breast cancer by the age of 29 years, and she died from metastatic cancer 6 months after diagnosis. This patient also had juvenile-onset diabetes as an additional complication, eventually accelerating the progression of the disease. Another patient, and her mother, who also had bilateral breast cancer, were heterozygotes for the 4772delA mutation in the *BRCA2* gene. The younger patient had her first cancer by the age of 54 years and a contralateral breast tumour by the age of 59, while her mother developed synchronous bilateral breast cancer by the age of 73 years as well as a histologically distinct recurrence in her first breast; she died from her cancer by the age of 79 years (Table 2). The other 3 *BRCA1* or *BRCA2* mutation carriers had developed metachronous contralateral cancer after time intervals of 2 y, 4.5 y and 12.5 y, respectively (in comparison with a median

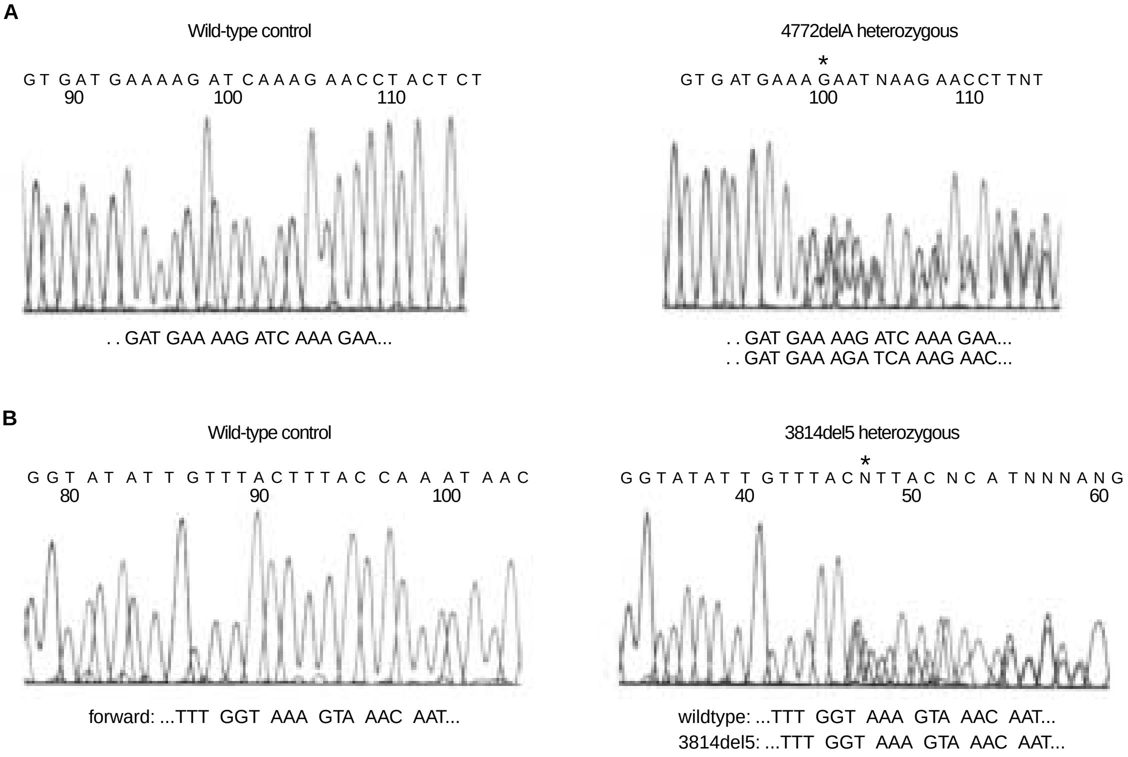

**Figure 1** Identification of new truncating mutations in the *BRCA1* and *BRCA2* genes. **(A)** Direct sequencing of BRCA2 mutation 4772delA in a patient with bilateral breast cancer. Left: wildtype control, right: heterozygous mutation carrier. An asterisk marks the start of the frameshift. **(B)** Direct sequencing of BRCA1 mutation 3814del5 in a patient with unilateral breast cancer. The antisense strand is shown. Left: wildtype control, right: heterozygous mutation carrier. An asterisk marks the start of the frameshift

 © 2001 Cancer Research Campaign interval of 7.2 y in the total cohort). In 3 of the 6 carriers with bilateral cancer, one of both tumours was multifocal. The family history of cancer in 1st and 2nd degree relatives was negative for 3 of the 5 unrelated patients who had bilateral cancer and a *BRCA1* or *BRCA2* truncating mutation.

Apart from these clearly pathogenic mutations, a further 11 amino acid variants and polymorphisms were found in the coding region of the *BRCA1* gene and 12 were detected in the *BRCA2* gene (Table 3). Common alterations with more than one occurrence were observed also in the comparison group, and their allele frequencies were not significantly different between the patients with bilateral breast cancer and the patients with unilateral breast cancer. There was no significant deviation in the genotype frequences from those expected for Hardy–Weinberg equilibrium. 7 polymorphisms of the *BRCA1* gene (2201C/T, 2430T/C, Pro871Leu, Glu1038Gly, Lys1183Arg, 4426C/T, Ser1613Gly) and 3 polymorphisms of the *BRCA2* gene (Asn289His, 1593A/G and Asn991Asp) were in absolute linkage disequilibrium, in accordance with previous findings in other populations (Durocher et al, 1996; Dunning et al, 1996; Southey et al, 1999; Wagner et al, 1999). A slightly higher prevalence of the Gln356Arg substitution was observed among patients with bilateral cancer (9/75 vs. 6/75, including one patient with unilateral breast cancer carrying the Cys61Gly mutation in cis with the Arg356 allele), a non-significant trend which may deserve further attention in larger cohorts taking into account that the Arg356 substitution is located within the ZBRK1-binding region and has recently been described to impair the *BRCA1*-mediated induction of GADD45 expression (Zheng et al, 2000). In addition, 2 unclassified missense variants of *BRCA2* (Pro1819Ser, Lys2950Asn) were found only in the cohort with bilateral disease, which can not be adequately addressed for their functional impact at the present time. Altogether, despite these subtle tendencies, the number and frequencies of missense substitutions did not differ significantly between the 2 cohorts of unilateral and bilateral breast cancer patients.

### DISCUSSION

Contralateral breast cancer risk appears to be strongly increased in multi-case families with a genetic predisposition through *BRCA1* and *BRCA2* gene mutations (Ford et al, 1994, 1998; Easton et al, 1995; Robson et al, 1998; Verhoog et al, 1998). These may act in concert with environmental factors such as radiation (Goss and Serra, 1998; Roberts et al, 1999), as there is accumulating evidence to implicate the *BRCA1* and *BRCA2* gene products in the cellular responses to radiation-induced damage (for review see Kote-Jarai and Eeles, 1999; Haber, 2000; Welcsh et al, 2000). It thus seems on solid biochemical grounds to assume that *BRCA1* and *BRCA2* mutations predispose towards the development of bilateral breast cancer via their impairment of DNA repair. On the other hand, the first population-based studies of a limited number of *BRCA1* and *BRCA2* gene mutations have revealed mutations only in a small minority of patients with bilateral disease, and most of these also had an early onset at first primary (Eccles et al, 1998; Gershoni-Baruch et al, 1999).

To gain more insight into the proportion of bilateral breast cancer that can be explained by *BRCA1* and *BRCA2* mutations in a hospital setting, we analysed the whole *BRCA1* and *BRCA2* coding region in 75 consecutive patients with bilateral breast cancer not selected for age or family history. Somewhat unexpectedly, only 5 unrelated carriers of a truncating mutation were identified. Family history would not have been a good indicator for *BRCA1* or *BRCA2* testing in these cases. Our finding that 3 unrelated patients and one mother–daughter pair with bilateral breast cancer had frameshift deletions in their *BRCA2* genes, does not support previous suggestions that *BRCA2* mutation carriers may be less prone to develop a contralateral cancer than *BRCA1* mutation carriers (Verhoog et al, 1999, 2000). However, in view that 4 of the 5 *BRCA2* deletion carriers developed their contralateral breast cancer after a first diagnosis at an age >45 years, it is possible that the age at first primary could be less influential in case of *BRCA2* mutations than it is for *BRCA1* gene mutations (Verhoog et al, 2000). The higher number of *BRCA2* carriers among patients with bilateral cancer compared with *BRCA1* carriers among the patients with unilateral breast cancer may deserve further investigation but could also reflect a maldistribution by chance due to the small numbers involved.

The proportion of 6.7% *BRCA1* and *BRCA2* deletion carriers among the patients with bilateral breast cancer in our study lies well within the range of 5–10% of breast cancer cases that are generally assumed to be caused by a strong familiar predisposition (Kote-Jarai and Eeles, 1999; Martin and Weber, 2000). 3 carriers (4%) were found also in the comparison group indicating that the prevalence of ‘classical’ deleterious *BRCA1* and *BRCA2* mutations is not significantly different in patients with bilateral and unilateral disease from the same department matched by age and family history. We thus investigated whether more common missense substitutions of *BRCA1* or *BRCA2* might play a role as predisposing factors towards contralateral cancer. Missense substitutions in other tumour suppressor genes have been described as low-penetrance mutations that may predispose to certain malignancies or modify disease progression (Larson et al, 1998; Otterson et al, 1999; Varley et al, 1999; Lumin et al, 2000; Rebbeck et al, 2000), and a few missense variants of the BRCA1 and BRCA2 proteins, including the Gln356Arg, Arg841Trp and Leu871Pro substitutions of *BRCA1* and the Asn372His substitution of *BRCA2*, have previously been implicated in the susceptibility to breast or ovarian cancer (Barker et al, 1996; Durocher et al, 1996; Dunning et al, 1996; Healey et al, 2000). However, none of the common *BRCA1* and *BRCA2* variants occurred at a significantly increased prevalence in our patients with bilateral cancer, nor did we find a deviation of the genotype distributions from Hardy–Weinberg equilibrium. Hence, there was no evidence to support a strong predisposition by any of the common missense substitutions. Our study had 80% power to detect relative risks of 2.5 or greater for polymorphisms with an allele frequency of about 0.3, therefore more subtle effects or moderate risks for less frequent variants cannot be finally excluded.

Altogether, these results suggest that patients with bilateral breast cancer, if not selected for age and family history, are not significantly more likely to be carriers of a *BRCA1* or *BRCA2* alteration than patients with unilateral breast cancer. In fact, the proportion of detected *BRCA1* and *BRCA2* mutations among patients with bilateral disease appeared much lower than would have been calculated from high-penetrance breast cancer families (Ford et al, 1994, 1998; Easton et al, 1995). If, for example, a *BRCA1* or *BRCA2* mutation confers a risk of about 50% to develop a contralateral breast cancer by the age of 54, we might have expected that about 1 in 4 patients with bilateral breast cancer in a similarly aged population-based cohort were carriers of a *BRCA1* or *BRCA2* mutation, a proportion to be detected with 80% power in our study. Instead, our present data are more consistent with an odds ratio of 1.7 (95% CI 0.4–5.0) in favour of *BRCA1* or *BRCA2*

© 2001 Cancer Research Campaign 

**Table 2** Clinical characteristics of breast cancer patients carrying *BRCA1* or *BRCA2* mutations

| | Mutation | Age at diagnosis | Family history (1° and 2° relatives) | Histology | Treatment | Course of disease | Follow up |
|---|---|---|---|---|---|---|---|
| Bilateral breast cancer | | | | | | | |
| 1 | BRCA 1<br>517delTG | 46 ys and 48 ys | none | BC 1: multifocal invasive-ductal,EIC<br><br>recurr. BC 1: invasive-ductal<br>BC 2: invasive-ductal | BCT: BC 1 and 2<br>CTX:BC 2<br>HT: BC 1<br>RT: BC 1 and 2 | BC 1: local failure after 25 mo. | 42 mo. |
| 2a | BRCA2<br>4772delA | 54 ys and 59 ys | BC: 1 (bilat., 2b) | BC 1: invasive-ductal<br>BC 2: invasive-ductal | BCT: BC 1 and 2<br>RT: BC 1 and 2<br>HT: BC 1 and 2 | NED | 17 mo. |
| 2b | BRCA2<br>4772delA | 73 ys (synchr.) | BC: 1 (bilat., 2a) | BC 1: invasive-lobular<br>recurr. BC1: invasive-ductal<br>BC 2: invasive-tubular | mastectomy: BC 1<br>BCT: BC 2<br>RT: BC 1, BC 2<br>and recurr. BC 1<br>HT: BC 1, BC 2,<br>and recurr. BC 1 | BC 1: local failure after 64 mo.<br>distant failure after 70 mo. | 76 mo.,<br>deceased |
| 3 | BRCA 2<br>5946delCT | 63 ys and 65 ys | none | BC 1: microinvasive-ductal<br>BC 2: invasive-ductal | BCT: BC 1 and 2<br>RT: BC 1 and 2<br>HT: BC 1 and 2 | NED | 9 mo. |
| 4 | BRCA 2<br>6174delT | 29 ys (synchr.) | none | BC 1: multifocal invasive-ductal<br>BC 2: invasive-ductal | BCT: BC 1 and 2<br>CTX<br>RT: BC 1 and 2 | distant failure after 4 mo. | 6 mo.,<br>deceased |
| 5 | BRCA 2<br>8138del5 | 46 ys and 59 ys | BC: 2 | BC 1: invasive–lobular<br>recurr.: invasive-ductal<br>BC 2: multifocal tubular | BCT: BC 1 and 2<br>RT: BC 1 and 2<br>HT: BC 1 and 2 | BC 1: local failures after 43<br>mo.,<br>57 mo. and 133 mo. | 10 mo. |
| Unilateral brest cancer | | | | | | | |
| 6 | BRCA 1 Cys61 Gly | 49 ys | OC: 1 | medullary | BCT, CTX, RT | NED | 45 mo. |
| 7 | BRCA 1<br>3814del5 | 54 ys | BC: 3 | multifocal invasive-ductal | mastectomy, CTX,<br>HT, RT (after local failure) | local failure<br>after 9 mo. | 24 mo.,<br>deceased |
| 8 | BRCA 1 5382insC | 42 ys | BC: 1 (bilat.) | invasive-ductal | BCT, CTX, RT | NED | 49 mo. |

Abbreviations: BCT = breast conservative therapy; RT = radiotherapy; CTX = chemotherapy, HT = hormonal therapy; BC = breast cancer; OC = ovarian cancer; NED = no evidence of disease, EIC = extensive intraductal component. Patients 2a and 2b are daughter and mother, respectively.

© 2001 Cancer Research Campaign

**Table 3** Polymorphisms and rare amino acid substitutions of *BRCA1* and *BRCA2*

| Gene | Exon | Codon | Amino acid substitution | Nucleotide | Nucleotide | Allele frequency no./total (rel. fraction) | |
|---|---|---|---|---|---|---|---|
| | | | | | | Bilateral | Unilateral |
| *BRCA1* | 11 | 356 | Gln/Arg | 1186 | CAG-CGG | 9/150 (0.06) | 6/150 (0.04) |
| | 11 | 694 | none | 2201 | AGC-AGT | 56/150 (0.37) | 52/150 (0.35) |
| | 11 | 771 | none | 2430 | TTG-CTG | 56/150 (0.37) | 52/150 (0.35) |
| | 11 | 871 | Pro/Leu | 2732 | CCG-CTG | 56/150 (0.37) | 52/150 (0.35) |
| | 11 | 1038 | Glu/Gly | 3232 | GAA-GGA | 56/150 (0.37) | 52/150 (0.35) |
| | 11 | 1040 | Ser/Asn | 3238 | AGC-AAC | 3/150 (0.02) | 3/150 (0.02) |
| | 11 | 1183 | Lys/Arg | 3667 | AAA-AGA | 56/150 (0.37) | 52/150 (0.35) |
| | 11 | 1347 | Arg/Gly | 4158 | AGA-GGA | 2/150 (0.01) | 3/150 (0.02) |
| | 13 | 1435 | none | 4426 | TCT-TTT | 56/150 (0.37) | 52/150 (0.35) |
| | 15 | 1512 | Ser/Ile | 4655 | AGT-ATT | 1/150 (0.01) | 2/150 (0.01) |
| | 16 | 1613 | Ser/Gly | 4956 | AGT-GGT | 56/150 (0.37) | 52/150 (0.35) |
| *BRCA 2* | 10 | 289 | Asn/His | 1093 | AAT-CAT | 6/150 (0.04) | 9/150 (0.06) |
| | 10 | 372 | Asn/His | 1342 | AAT-CAT | 43/150 (0.29) | 42/150 (0.28) |
| | 10 | 455 | none | 1593 | TCA-TCG | 6/150 (0.04) | 9/150 (0.06) |
| | 11 | 991 | Asn/Asp | 3199 | AAC-GAC | 6/150 (0.04) | 9/150 (0.06) |
| | 11 | 1132 | none | 3624 | AAA-AAG | 45/150 (0.30) | 32/150 (0.21) |
| | 11 | 1172 | Ser/Leu | 3742 | TCG-TTG | 2/150 (0.01) | 1/150 (0.01) |
| | 11 | 1269 | none | 4035 | GTT-GTC | 28/150 (0.19) | 26/150 (0.17) |
| | 11 | 1819 | Pro/Ser | 5683 | CCC-TCC | 1/150 (0.01) | 0/150 |
| | 11 | 1915 | Thr/Met | 5973 | ACG-ATG | 4/150 (0.03) | 2/150 (0.01) |
| | 14 | 2414 | none | 7470 | TCA-TCG | 24/150 (0.16) | 23/150 (0.15) |
| | 18 | 2728 | Val/Ile | 8410 | GTT-ATT | 1/150 (0.01) | 1/150 (0.01) |
| | 22 | 2950 | Lys/Asn | 9078 | AAG-AAT | 1/150 (0.01) | 0/150 |

Codon and nucleotide positions were designated according to the BRCA1 and BRCA2 cDNA sequences. Nucleotide substitutions are shown within their codon context. Allele frequencies are given as allelic counts out of the total chromosomes and as relative frequencies (in brackets). Haplotype or genotype frequencies were not significantly different between both groups for either of the substitutions (see text).

mutation carriers in bilateral breast cancer. Although the confidence intervals are wide and the results have to be confirmed with larger sample sizes, the identification of only 5 mutation carriers among 75 patients with bilateral breast cancer suggests that the carrier frequency in our hospital-based sample may be about two- or threefold lower than expected from multi-case family studies. An important difference between population-based association studies and family-based studies lies in the fact that the magnitude of relative risks in the latter is conditional on shared genetic and environmental influences. It is therefore possible that the high frequency of bilateral disease in multi-case breast cancer families may be due to a familial aggregation of other susceptibility factors modifying the penetrance of *BRCA1* and *BRCA2* mutations. Such a view would be supported by the recent observation that within *BRCA1* families the contralateral breast cancer risk is also associated with the age at onset of the primary tumour (Verhoog et al, 2000).

These apparent differences between family-based and population-based studies may have implications for therapeutic decision making. For instance, it has been proposed that the magnitude of life expectancy gains from cancer prevention strategies, including prophylactic contralateral mastectomy, strongly depends on the penetrance of the *BRCA* mutation (Schrag et al, 2000). Our results suggest that the penetrance with regard to contralateral disease is – at least in our hospital-based sample – not necessarily a feature determined only by the nature or location of the *BRCA* mutation itself, as the mutational types and frequencies were similar in our patients with unilateral and bilateral breast cancer. There may be additional, yet undetermined, factors that influence the contralateral breast cancer risk. Some of the known putative genetic modifiers include ATM mutations, rare H-ras alleles or androgen receptor gene variations (Phelan et al, 1996; Larson et al, 1998; Rebbeck et al, 1999; Broeks et al, 2000). We have presently no evidence that bilaterality of breast cancer alone were a strong indicator of *BRCA1* and *BRCA2* mutations if adjusted for age and family history.

### ACKNOWLEDGEMENTS

We wish to thank Andrea Korte, Hildegard Frye and PD Dr Manfred Stuhrmann for their support with DNA extractions. Part of this work was funded by a grant from the Medical School Hannover to MB and TD.

### REFERENCES

Adami HO, Bergstrom R and Hansen J (1985) Age at first primary as a determinant of the incidence of bilateral breast cancer. Cumulative and relative risks in a population-based case–control study. *Cancer* **55**: 643–647

Anderson DE (1971) Some characteristics of familial breast cancer. *Cancer* **28**: 1500–1505

Barker DF, Almeida ERA, Casey G, Fain PR, Liao S-Y, Masunaka I, Noble B, Kurosaki T and Anton-Culver H (1996) *BRCA1* R841W: A strong candidate for a common mutation with moderate phenotype. *Genet Epidemiol* **13**: 595–604

Beckmann MW, Niederacher D, Goecke TO, Bodden-Heinrich R, Schnürch H-G and Bender HG (1997) Hochrisikofamilien mit Mamma-und Ovarialkarzinomen. Möglichkeiten der Beratung, genetischen Analyse und Früherkennung. *Dt Ärztebl* **94**: A161–167

Bernstein JL, Thompson WD, Risch N and Holford TR (1992) The genetic epidemiology of second primary breast cancer. *Am J Epidemiol* **136**: 937–948

Broeks A, Urbanus JHM, Floore AN, Dahler EC, Klijn JGM, Rutgers EJT, Devilee P, Russell NS, van Leeuwen FE and van't Veer L (2000) ATM-heterozygous germline mutations contribute to breast cancer-susceptibility. *Am J Hum Genet* **66**: 494–500

© 2001 Cancer Research Campaign

Dunning AM, Chiano M, Smith NR, Dearden J, Gore M, Oakes S, Wilson C, Stratton M, Peto J, Easton D, Clayton C and Ponder BAJ (1996) Common BRCA1 variants and susceptibility to breast and ovarian cancer in the general population. *Hum Mol Genet* **6**: 285–289

Durocher F, Shattuck-Eidens D, McClure M, Labrie F, Skolnick MH, Goldgar DE and Simard J (1996) Comparison of BRCA1 polymorphisms, rare sequence variants and/or missense mutations in unaffected and breast/ovarian cancer populations. *Hum Mol Genet* **5**: 835–842

Easton DF, Ford D, Bishop DT, and the Breast Cancer Linkage Consortium (1995) Breast and ovarian cancer incidence in *BRCA1*-mutation carriers. *Am J Hum Genet* **56**: 265–271

Eccles DM, Englefield P, Soulby MA and Campbell IG (1998) BRCA1 mutations in southern England. *Br J Cancer* **77**: 2199–2203

Ford D, Easton DF, Bishop DT, Narod SA, Goldgar DE, and the Breast Cancer Linkage Consortium (1994) Risks of cancer in BRCA1 – mutation carriers. *Lancet* **343**: 692–695

Ford D, Easton D and Stratton M, et al., for the Breast Cancer Linkage Consortium (1998) Genetic heterogeneity and penetrance analysis of the *BRCA1* and *BRCA2* genes in breast cancer families. *Am J Hum Genet* **62**: 676–689

Gershoni-Baruch R, Dagan E, Fried G, Kepten I and Robinson E (1999) BRCA1 and BRCA2 founder mutations in patients with bilateral breast cancer. *Eur J Hum Genet* **7**: 833–836

Goss PE and Sierra S (1998) Current perspectives on radiation-induced breast cancer. *J Clin Oncol* **16**: 338–347

Haber D (2000) Roads leading to breast cancer. *N Engl J Med* **343**: 1566–1568

Healey CS, Dunning AM, Dawn Teare M, Chase D, Parker L, Burn J, Chang-Claude J, Mannermaa A, Kataja V, Huntsman DG, Pharoah PDP, Luben RN, Easton DF and Ponder BAJ (2000) A common variant in BRCA2 is associated with both breast cancer risk and prenatal viability. *Nature Genet* **26**: 362–364

Healey EA, Cook EF, Orav EJ, Schnitt SJ, Connolly JL and Harris JR (1993) Contralateral breast cancer: clinical characteristics and impact on prognosis. *J Clin Oncol* **11**: 1545–1552

Hislop TG, Elwood JM, Coldmann AJ, Spinelli JJ, Wort JJ and Ellison LG (1984) Second primary cancer of the breast: incidence and risk factors. *Br J Cancer* **49**: 70–85

Knudson AG Jr (1971) Mutation and cancer: Statistical study of retinoblastoma. *Proc Natl Acad Sci USA* **68**: 820–823

Kote-Jarai Z, Eeles RA (1999) BRCA1, BRCA2 and their possible function in DNA damage response. *Br J Cancer* **81**: 1099–1102

Lamlum H, Tassan NA, Jaeger E, Frayling I, Sieber O, Bin Reza F, Eckert M, Rowan A, Barclay E, Atkin W, Williams C, Gilbert J, Cheadle J, Bell J, Houlston R, Bodmer W, Sampson J and Tomlinson I (2000) Germline *APC* variants in patients with multiple colorectal adenomas, with evidence for the particular importance of E1317Q. *Hum Mol Genet* **9**: 2215–2221

Larson GP, Zhang G, Ding S, Foldenauer KF, Udar N, Gatti RA, Neuberg D, Lunetta KL, Ruckdeschel JC, Longmate J, Flanagan S and Krontiris TG (1998) An allelic variant at the ATM locus is implicated in breast cancer susceptibility. *Genet Testing* **1**: 165–170

Martin A-M and Weber BL (2000) Genetic and hormonal risk factors in breast cancer. *J Natl Cancer Inst* **92**: 1126–1135

Mose S, Adamietz IA, Thilmann C, Saran F, Pahnke R and Böttcher HD (1995) Die Prognose des bilateralen Mammakarzinoms im Vergleich zum unilateralen Mammatumor. *Strahlenther Onkol* **171**: 207–213

Olsen JH, Seersholm N, Boice JD, Kruger Kjaer S and Fraumeni JF (1999) Cancer risk in close relatives of women with early-onset breast cancer – a population-based incidence study. *Br J Cancer* **79**: 673–679

Otterson GA, Modi S, Nguyen K, Coxon AB and Kaye FJ (1999) Temperature-sensitive RB mutations linked to incomplete penetrance of familial retinoblastoma in 12 families. *Am J Hum Genet* **65**: 1040–1046

Peto J and Mack TM (2000) High constant incidence in twins and other relatives of women with breast cancer. *Nature Genet* **26**: 411–414

Phelan CM, Rebbeck TR, Weber BL, Devilee P, Ruttledge MH, Lynch HT, Lenoir GM, Stratton MR, Easton DF, Ponder BAJ, Cannon-Albright L, Larsson C, Goldgar DE and Narod SA (1996) Ovarian cancer risk in BRCA1 carriers is modified by the HRAS1 variable number of tandem repeat (VNTR) locus. *Nature Genet* **12**: 309–311

Phillips KA, Glendon G and Knight JA (1999) Putting the risk of breast cancer in perspective. *N Engl J Med* **340**: 141–144

Rebbeck TR, Kantoff PW, Krithivas K, Neuhausen S, Blackwood MA, Godwin AK, Daly MB, Narod SA, Garber JE, Lynch HT, Weber BL and Brown M (1999) Modification of BRCA1 – associated breast cancer risk by the polymorphic androgen-receptor CAG repeat. *Am J Hum Genet* **64**: 1371–1377

Rebbeck TR, Walker AH, Zeigler-Johnson C, Weisburg S, Martin A-M, Nathanson KL, Wein AJ and Malkowicz SB (2000) Association of HPC2/ELAC2 genotypes and prostate cancer. *Am J Hum Genet* **67**: 1014–1019

Roberts SA, Spreadborough AR, Bulman B, Barber JBP, Evans DGR and Scott D (1999) Heritability of cellular radiosensitivity: a marker of low-penetrance predisposition genes in breast cancer? *Am J Hum Genet* **65**: 784–794

Robson M, Gilewki T and Haas B (1998) BRCA-associated breast cancer in young women. *J Clin Oncol* **16**: 1642–1649

Schrag D, Kuntz KM, Garber JE and Weeks JC (2000) Life expectancy gains from cancer prevention strategies for women with breast cancer and *BRCA1* or *BRCA2* mutations. *JAMA* **283**: 617–624

Southey MC, Tesoriero AA, Anderson CR, Jennings KM, Brown SM, Dite GS, Jenkins MA, Osborne RH, Maskiell JA, Porter L, Giles GG, McCredie MRE, Hopper JL and Venter DJ (1999) *BRCA1* mutations and other sequence variants in a population-based sample of Australian women with breast cancer. *Br J Cancer* **79**: 34–39

Varley JM, McGown G, Thorncroft M, James LA, Margison GP, Forster G, Evans DGR, Harris M, Kelsey AM and Birch JM (1999) Are there low-penetrance p53 alleles? Evidence from childhood adrenocortical tumors. *Am J Hum Genet* **65**: 995–1006

Verhoog LC, Brekelmans CTM and Seynaeve C (1998) Survival and tumour characteristics of breast-cancer patients with germline mutations of BRCA1. *Lancet* **251**: 316–321

Verhoog LC, Brekelmans CTM and Seynaeve C, Dahmen G, van Geel AN, Bartels CCM, Tilanus-Linthorst MMA, Wagner A, Devilee P, Halley DJJ, van den Ouweland AMW, Meijers-Heijboer EJ and Klijn JGM (1999) Survival in hereditary breast cancer associated with germline mutations of BRCA2. *J Clin Oncol* **17**: 3396–3402

Verhoog LC, Brekelmans CTM, Seynaeve C, Meijers-Heijboer EJ and Klijn JGM (2000) Contralateral breast cancer risk is influenced by the age at onset in BRCA1-associated breast cancer. *Br J Cancer* **83**: 384–386

Wagner TMU, Hirtenlehner K, Shen P, Moeslinger R, Muhr D, Fleischmann E, Concin H, Doeller W, Haid A, Lang AH, Mayer P, Petru E, Ropp E, Langbauer G, Kubista E, Scheiner O, Underhill P, Mountain J, Stierer M, Zielinski C and Oefner P (1999) Global sequence diversity of *BRCA2*: analysis of 71 breast cancer families and 95 control individuals of worldwide populations. *Hum Molec Genet* **8**: 413–423

Welcsh PL, Owens KN and King M-C (2000) Insights into the functions of BRCA1 and BRCA2. *Trends Genet* **16**: 69–74

Zheng L, Pan H, Li S, Flesken-Nikitin A, Chen P-L, Boyer TG and Lee W-H (2000) Sequence-specific transcriptional corepressor function for BRCA1 through a novel zinc finger protein, ZBRK1. *Molecular Cell* **6**: 757–768

 *© 2001 Cancer Research Campaign*

### APPENDIX

**Table A1** Primers used for the genomic PCR amplification of *BRCA1* and *BRCA2* gene segments

| Exon | Forward primer | Reverse primer | Annealing (°C) | Size (bp) |
|---|---|---|---|---|
| *BRCA1* | | | | |
| 2 | GTGTTAAAGTTCATTGGAACAG | GCATAGGAGATAATCATAGGAATCC | 58 | 162 |
| 3 | TGAGGCCTTATGTTGACTCAG | CTGGGTTATGAAGGACAAAAAC | 62 | 220 |
| 5 | CTCTTAAGGGCAGTTGTGAG | TTCCTACTGTGGTTGCTTCC | 64 | 234 |
| 6 | GGTTGATAATCACTTGCTGAG | GCAAACTTCCTGAGTTTTCATG | 62 | 200 |
| 7 | GCATACATAGGGTTTCTCTTGG | AGAAGAAGAAGAAAACAAATGG | 59 | 258 |
| 8 | CTGGCCAATAATTGCTTGACTG | CTATAAGATAAGGAATCCAGC | 60 | 207 |
| 9 | GTTGAATATCTGTTTTTCAAC | CCAGCTTCATAGACAAAGGTTC | 62 | 172 |
| 10 | TTGACAGTTCTGCATACATG | AGGTCCCAAATGGTCTTCAG | 62 | 232 |
| 11/F | CACCTCCAAGGTGTATGAAG | CTCTAGGATTCTCTGAGCATGG | 58 | 506 |
| 11/1 | CCAAAGCGAGCAAGAGAATCTC | ATGAGTTGTAGGTTTCTGCTGTG | 63 | 1337 |
| 11/2 | ACAATTCAAAAGCACCTAAAAAG* | AACCCCTAATCTAAGCATAGCATTC | 63 | 1463 |
| 11/3 | CACCACTTTTTCCCATCAAGTC* | TTATTTTCTTCCAAGCCCGTTCC | 63 | 1123 |
| 11/R | CTACTAGGCATAGCACCGTTGC | CACTTCTATAAATAGACTGGG | 58 | 419 |
| 12 | AAGACACAGCAAGTTGCAGCG | GGGATACATACTACTGAATGC | 64 | 224 |
| 13 | TAATGGAAAGCTTCTCAAAG | TCCTTACTCTTCAGAAGGAG | 58 | 306 |
| 14 | TCACTATCAGAACAAAGCAG | GATGTCAGATACCACAGCATC | 58 | 220 |
| 15 | TGCCCAGCAAGTATGATTTG | TTGTTCCAATACAGCAGATG | 59 | 289 |
| 16 | CTTAACAGAGACCAGAACTTTG | GTCATTAGGGAGATACATATGG | 58 | 415 |
| 17 | GTGTGCTAGAGGTAACTCATG | GCAGCAGATGCAAGGTATTC | 58 | 177 |
| 18 | TCTTAGGACAGCACTTCCTG | CTGAGGTGTTAAAGGGAGGAG | 64 | 190 |
| 19 | AGCACGTTCTTCTGCTGTATG | TCTGGTTAGTTTGTAACATC | 58 | 120 |
| 20 | TCTCTTATCCTGATGGGTTG | GGGAATCCAAATTACACAGC | 62 | 338 |
| 21 | CATCAGGTGGTGAACAGAAG | GTAAGACAAAGGCTGGTGCTG | 64 | 243 |
| 22 | GGTAGAGGGCCTGGGTTAAG | CAGGTGCCAGTCTTGCTCAC | 64 | 208 |
| 23 | TCCAGTAGTCCTACTTTGAC | ACCCCATATAGCACAGGTAC | 60 | 149 |
| 24 | GATTGATTAGAGCCTAGTCCAGG | CAGCCTGAATAGAAAGAATAGGGC | 64 | 519 |
| *BRCA2* | | | | |
| 2 | TATTTACCAAGCATTGGAGG | TAGAAAACACTTTCTCGGTG | 64 | 156 |
| 3 | ACAAATTTGTCTGTCACTGG | GTAGTTCTCCCCAGTCTACC | 64 | 338 |
| 4 | TCATTCCCAGTATAGAGGAG | CTTCTACCAGGCTCTTAGCC | 59 | 329 |
| 5/6 | AAGATAAACTAGTTTTTGCCAG | GGGCAAAGGTATAACGCTATTGTC | 56 | 328 |
| 7 | ATATCCTTAATGATCAGGGC | GAGATGACAATTATCAACCTC | 59 | 231 |
| 8 | GTAATCAAATAGTAGATGTGC | TGTTAGCAATTTCAACAGTC | 59 | 190 |
| 9 | AAGTGAAACCATGGATAAGGG | CACGGGAGGCAGAGGTTGCGG | 61 | 322 |
| 10/F | TCTATGAGAAAGGTTGTGAG | CAAAGACGGTACAACTTCCTTG | 59 | 418 |
| 10 | TATACTTTAACAGGATTTGG*° | ACACAGAAGGAATCGTCATC | 53 | 1116 |
| 11/F | CTTCCTGCCTCAGCCTCCCAAAAG | GCTAGTTAAGGACAAAGTTGG | 58 | 154 |
| 11/1 | TTTTTATGTTTAGGTTTATT*° | TGCATTCCTCAGAAGTGGTC | 57 | 1569 |
| 11/2 | AAACCAAGCTACATATTGC*° | TAATTTCCTACATAATCTGCAG | 59 | 1751 |
| 11/3 | TGGCTTAGAGAAGGAATAT*° | AAAATAGTGATTGGCAACACG | 63 | 1769 |
| 12 | AATGGTCTATAGACTTTTGAG | ATCCATACCTATAGAGGGAG | 59 | 218 |
| 13 | ACAGTAACATGGATATTCTC | ACGAGACTTTTCTCATACTG | 59 | 187 |
| 14 | TGAGGGTCTGCAACAAAGGC | CAGGACATTATTTAACAACGG | 64 | 573 |
| 15 | GTGCCTGGCCAGGGGTTGTGC | CTCTGTCATAAAAGCCATCAG | 60 | 281 |
| 16 | ATTGTGTGATACATGTTTAC | GTTCGAGAGACAGTTAAGAG | 59 | 322 |
| 17 | TTCAGTATCATCCTATGTGG | TACTGCCGTATATGATTACG | 59 | 267 |
| 18 | ACAGTGGAATTCTAGAGTCAC | TTTAACTGAATCAATGACTG | 56 | 460 |
| 19 | AAGGCAGTTCTAGAAGAATG | AAGAGACCGAAACTCCATATC | 54 | 339 |
| 20 | CCACTGTGCCTGGCCTGATAC | GTCTCTAAGACTTTGTTCTC | 64 | 283 |
| 21 | CTTTGGGTGTTTTATGCTTG | CTTGAATAATCATCAAGCCTC | 59 | 251 |
| 22 | CCATCTAGTTACAATAGATG | ATCATTTTGTTAGTAAGGTC | 54 | 339 |
| 23 | GATAATCACTTCTTCCATTGC | TAAACTAACAAGCACTTATC | 54 | 260 |
| 23/24 | GATAATCACTTCTTCCATTGC | AACTGGTAGCTCCAACTAATC | 64 | 531 |
| 25 | GGCATATTAGAGTTTCCTTTC | CAAAATGTGTGGTGATGCTG | 64 | 361 |
| 26 | CATCGGCATGTTTGACAATTGG | CATTTTTACCATGTTTACTAGG | 56 | 390 |
| 27 | TAGGGGAGGGAGACTGTGTG | AACTGGAAAGGTTAAGCGTC | 64 | 779 |

*/*° A T7 promotor and a eukaryotic translation initiation sequence with an ATG start codon were added at the 5′-end for further use of this primer in a protein truncation test.

© 2001 Cancer Research Campaign 

**Table A2** Duplex PCR conditions

| Gene/Exon | Annealing (°C) | Primer ratio |
|---|---|---|
| BRCA 1/9 + 20 | 60 | 3:1 |
| BRCA 2/2 + 3 | 62 | 1:1 |
| BRCA 2/4 + 12 | 57 | 1:1 |
| BRCA 2/7 + 8 | 59 | 1:1 |
| BRCA 2/13 + 17 | 57 | 1:1 |
| BRCA 2/16 + 21 | 57 | 1:1 |

**Table A3** Restriction fragments for SSCP analysis

| Gene/Exon | Restriction enzyme | Size of fragments (bp) |
|---|---|---|
| 1/11 F | *Eco*RI | 187/319 |
| 1/11/1 | *Alw* I, *Hph* I | 363/270/372/332 |
| 1/11/2 | *Dde* I | 238/207/225/306/383 |
| 1/11/3 | *Fok* I, *Eco* NI | 358/371/186/208 |
| 1/11R | *Eco*NI | 135/284 |
| 1/16 | *Mbo* I | 131/284 |
| 1/24 | *MboI* | 173/346 |
| 2/10F | *Nla* III | 171/289 |
| 2/10 | *Dpn* II, *Bsa*AI | 297/250/236/333 |
| 2/11/1 | *Hph* I, *Spe* I | 297/316/290/306/360 |
| 2/11/2 | *Pst* I, *Bcl* I, *Bsa* I, *Scr* FI | 366/318/273/169/216/389 |
| 2/11/3 | *Hph* I, *Bsa*AI | 379/384/272/175/273/294 |
| 2/14 | *Dra* I | 303/270 |
| 2/18 | *Dde* I | 290/170 |
| 2/23+24 | *Msp* I | 208/323 |
| 2/26 | *Dpn* II | 213/177 |
| 2/27 | *Drd* I/*Nla* IV/*Mbo* I | 218/169/257/133 |

**Table A4a** Restriction-enzyme based mutation screening assays

| Gene/Exon | Primer pair | Size (bp) | Annealing | Substitution | Enzyme | Fragments |
|---|---|---|---|---|---|---|
| BRCA1/5 | BR5F/BR5R | 234 | 64 | Cys61Gly | *Ava* II | Cys 234, Gly 154/80 |
| BRCA1/11 | BS1/BS2 | 1337 | 63°C | Gln356Arg | *Alw* NI | Gln 396/941, Arg 1337 |
| BRCA1/11 | BS5/BS4 | 325 | 59°C | Glu1038Gly | *Nla* IV | Glu 325, Gly 172/153 |
| BRCA1/11 | BS5/BS4 | 325 | 59°C | Ser1040Asn | *Alu* I | Ser 178/147, Asn 325 |
| BRCA1/11 | BS5B/R1347G | 341 | 60°C | Arg1347Glu | *Bsu*F5/*Eco* NI | Arg 189/152, Glu 189/134/18 |
| BRCA1/15 | BR15F2/BR15S-I | 145 | 64°C | Ser 1511 ile | *Alw* NI | Ser 145, ile 125/20 |
| BRCAI/16 | BR16F/BR16R | 415 | 58°C | Ser1613Gly | *Ava*II, *Scr* FI, *Nla* IV | Ser 415, Gly 179/236 |
| BRCA1/20 | BS9/BS10 | 270 | 60°C | 5382insC | *Scr*FI, | wildtype 270, mutant 248/22 |
| | | | | | *Dde*I | wildtype 214/22, mutant 236 |
| BRCA2/10 | BS11/1342D | 359 | 56°C | Asn372His | *Nla*III | Asn 359, His 334/25 |
| BRCA2/10 | BS11/1342D | 359 | 56°C | Asn289His | *Nla*III, *Afl* III | Asn 359, His 274/85 |
| BRCA2/11 | BS14/3624D | 111 | 54°C | A3624G | *Mwo*I | A 111, G 86/25 |
| BRCA2/11 | BS15/16 | 1751 | 59°C | Serl172Leu | *Taq* | Leu 1751, Ser 1630/121 |
| BRCA2/11 | BS16B/4035D | 361 | 56°C | T4035C | *Tth* IIII | T 361, C 336/25 |
| BRCA2/11 | BS19/18B | 436 | 59°C | Thr1915Met | *Nsi*I | Thr 436, Met 325/111 |
| BRCA2/11 | 6174delT/BS20A | 375 | 61°C | 6174delT | *Pfl* MI | mutant 375, wildtype 354/21 |
| BRCA2/18 | BR2-18F/V2728I-R | 286 | 60°C | Val2728*Ile* | HpaI | Val 265/21, Ile 286 |

**Table A4b** Sequences of mismatch primers used in restriction-enzyme based screening assays

| Gene/Exon | Gene alteration | Designation | Sequence (5′-3′, mismatch) | Use in Table 4a |
|---|---|---|---|---|
| BRCA1/11 | A4158G | R1347G-R | ATTTTCTTCCAAGCCCGTTCATC | Reverse primer |
| BRCA1/15 | G4654T | BR15-S/I | CTATTCTGAAGACTCCCACAGC | Reverse primer |
| BRCA1/20 | 5382insC | BS10 | CCAAAGCGAGCAAGAGAATCTC | Forward primer |
| BRCA2/10 | C1342A | 1342D | CTTCCACTCTCAAAGGGCTTCTCAT | Reverse primer |
| BRCA2/11 | A3624G | 3624D | GTCAGTTTGAATTTACTCAGCTTAG | Forward primer |
| BRCA2/11 | T4035C | 4035D | TCTTCAAGTTTTTGTCATGACTCTG | Forward primer |
| BRCA2/11 | 6174delT | 6174delT | GTGGGATTTTTAGCCCAGCAAG | Forward primer |
| BRCA2/18 | G8410A | V2728I-R | GGGAGGATCTAACTGGGCGTTAA | Reverse primer |

 *© 2001 Cancer Research Campaign*
